# Anti-neutrophil cytoplasmic antibody-associated hypertrophic cranial pachymeningitis and otitis media: a review of literature

**DOI:** 10.1007/s00405-018-5172-4

**Published:** 2018-10-17

**Authors:** Anquan Peng, Xinming Yang, Weijing Wu, Zian Xiao, Dinghua Xie, Shenglei Ge

**Affiliations:** Department of Otolaryngology-Head and Neck Surgery, The Second Xiangya Hospital, Central South University, Changsha, People’s Republic of China

**Keywords:** Anti-neutrophil cytoplasmic antibody, Vasculitis, Hypertrophic pachymeningitis, Otitis media

## Abstract

**Background and objective:**

It has been recognized that anti-neutrophil cytoplasmic antibody (ANCA)-associated vasculitides may lead to hypertrophic pachymeningitis (HP) or intractable otitis media (OM). To our knowledge, few cases of coexistent ANCA-related HP and OM have been described previously. To increase awareness of this disease, we reviewed the literature describing patients with HP and intractable OM in a population with AAV to guide clinical decision making for otolaryngologists.

**Methods:**

PubMed was searched with the following terms: ANCA-associated vasculitis, otitis media, and hypertrophic pachymeningitis. Only patients with concomitant AAV, OM and HP were considered and included in this review.

**Results:**

A total of 243 articles were reviewed, and of these, 6 met inclusion criteria. Headache, cranial polyneuropathy, and intractable OM with effusion or granulation were common. Serum MPO–ANCA positivity was most common in Asian patients. Almost all patients had dural mater thickening on gadolinium-enhanced magnetic resonance imaging of the brain. Corticosteroids plus an immunosuppressant was more effective and most patients had improved hearing after treatment, but approximately 50% of subjects had disease relapse.

**Conclusion:**

In this review, we summarized the current knowledge on the clinical features, diagnosis, treatment, and pathogenesis of this disease. We should carefully detect the potential cases of ANCA-related HP and OM in patients with intractable OM, HP, or AAV, and make the optimal treatment plan to avoid long-term neurological complications and irreversible hearing loss. Furthermore, due to an increased possibility of relapse, close follow-up, including a hearing test, ANCA titers, imaging examination, and detection of toxic and side effects of immunosuppressive therapy, are necessary.

## Introduction

Anti-neutrophil cytoplasmic antibody (ANCA)-associated vasculitis (AAV) is a rare necrotizing vasculitis with few or no immune deposits, predominantly affecting small vessels associated with ANCA specific for myeloperoxidase (MPO) or proteinase 3 (PR3). In small vessels, AAV includes granulomatosis with polyangiitis (Wegener’s granulomatosis) (GPA), microscopic polyangiitis (MPA), and eosinophilic granulomatosis with polyangiitis (EGPA) [1–5]. Recently, AAV has been shown to lead to hypertrophic pachymeningitis (HP), which accounts for most AAV cases. HP is a rare chronic disorder characterized by dural thickening. It is idiopathic or secondary to various conditions, such as infections (e.g., neurosyphilis or fungal meningitis), inflammatory disorders (e.g., rheumatoid arthritis or neurosarcoidosis), and neoplasms (e.g., dural carcinomatosis and meningioma). AAV is a major cause of HP, and ANCA-related HP (i.e. ANCA-associated HP) is the most common form among HP patients, representing as many as 34.0% of all HP cases in Japan [6]. In addition, otitis media (OM) may also co-occur with AAV. For example, 30–50% of GPA patients had OM, so and thus the concept of OM with AAV (OMAAV) was proposed to help understand the disease process better [7, 8]. We investigated whether ANCA-related HP and OM can occur simultaneously because Harabuchi’s group reported that 22.2% of ANCA-related HP patients had otologic symptoms, suggesting the presence of OM [8]. In addition, 17% of OMAAV patients were reported to have HP at their initial visit, and 28% have it at the end of the clinical course [6]. Therefore, few patients have ANCA-related HP and OM simultaneously. Some patients may require treatment for hearing loss, headache, with or without cranial nerve paralysis. Such cases are easily misdiagnosed, so better criteria are needed for early diagnosis and to avoid long-term neurological complications and irreversible hearing loss. To guide clinical decision making for otolaryngologists, we performed this review and tried to elucidate the characteristics of patients with HP and intractable OM in a population with AAV.

## Materials and methods

PubMed was searched for papers published between 1946 and May, 2018 addressing cases with ANCA-related HP and OM (i.e. AAV with HP and OM, ANCA-related HP and OM, or HP and OM plus AAV). A search strategy was developed with the following terms: HP, OM, AAV (electronic search: “hypertrophic pachymeningitis” AND “otitis media,” “Hypertrophic pachymeningitis” AND “ANCA-associated vasculitis,” “otitis media” AND “ANCA-associated vasculitis,” and “hypertrophic pachymeningitis” AND “otitis media” AND “ANCA-associated vasculitis”). Studies in English, Chinese, and Japanese were included, and full-text articles were reviewed. All patients included had AAV, HP, and intractable OM or they were excluded.

## Results

**Fig. 1:**
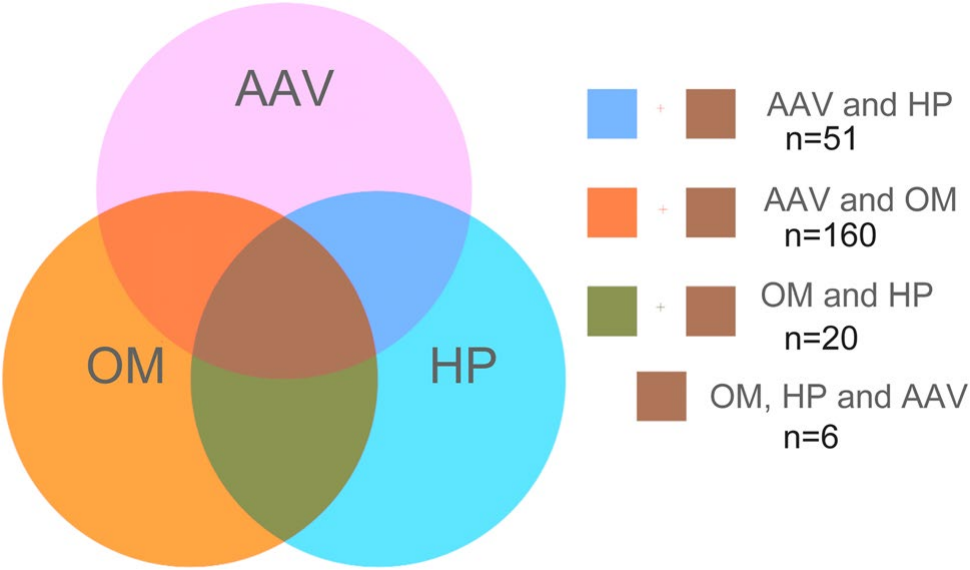
Search strategy scheme

We identified 243 articles but finally reviewed 20 studies describing HP associated with OM (Table 1) [8–27], and of these, six met inclusion criteria (Fig. 1). Some patient data likely overlapped among studies due to being from similar institutions or case sources. Most patients with coexistent ANCA-related HP and OM were elderly women. Headache, cranial polyneuropathy and otologic symptoms, such as hearing-loss, otorrhea, otalgia, and tinnitus, were common. Serum MPO–ANCA positivity was most common in Asian patients. All patients had dural mater thickening and enhancement on gadolinium-enhanced magnetic resonance imaging (MRI) of the brain. Treatment consisted of corticosteroid alone; corticosteroid plus immunosuppressant; and corticosteroid alone initially with immunosuppressant added after relapse. Most patients had hearing improvement after treatment, but for almost 50%, hearing loss returned. Relapse was less frequent for patients treated with corticosteroids and an immunosuppressant during their limited follow-up period.

**Table 1:** Summary of previously reported cases with HP and OM

Authors	Years	Age	Sex	Hearing loss	Headache	Facial paralysis	Cranial nerves or other sites involvement	Serum ANCA		MRI	Operation or biopsy	Primary treatment	Symptom improvement
								MPO–ANCA	PR3-ANCA				
Ishii et al. [9]	1991	55	F	Left MHL	ND	Left	+	ND	ND	+	Craniotomy	Prednisolone and antibiotics	Yes
Murai et al. [10]	1992	59	F	Bilateral MHL	+	Bilateral	ND	ND	ND	+	No	Miconazole, flucytosin and fluconazole (otorrhea culture: Aspergillus flavus)	Yes
Oku et al. [11]	1995	52	F	Right CHL	+	−	ND	ND	ND	+	Right suboccipital craniectomy	Corticosteroid	Yes
Adachi et al. [12]	1995	67	F	Right MHL	+	Left	+	ND	ND	+	Biopsy of the epidural mass of T5	Antibiotics	Yes
Yi et al. [13]	2000	26	M	Right MHL	+	Right	+	ND	ND	+	Mastoidectomy and labyrinthectomy	Antibiotics (otorrhea culture: Staphylococcus)	Yes
Kanzaki et al. [14]	2004	70	F	Bilateral MHL	ND	Bilateral	+	−	−	+	Mastoidectomy	Hydrocortisone and Vancomycin (otorrhea culture: MRSA)	Yes
Sato et al. [15]	2004	65	F	Right MHL	+	ND	+	ND	ND	+	ND	Antibiotics	Yes
Tada et al. [16]	2006	54	M	Bilateral SHL	+	ND	+	ND	ND	+	Biopsy of the dura matter, lung, skin and nasal mucosa	Corticosteroid and cyclophosphamide	Yes
*Iwasaki et al. [17]	2006	36	F	Left CHL	+	−	+	−	−	+	Craniotomy	Prednisolone	Yes
		54	M	Right CHL	+	Right	+	−	−	+	Craniotomy	Prednisolone	Yes
		67	M	Left CHL	+	−	+	−	+	+	No	Methylprednisolone and prednisolone	Yes
		54	F	Left MHL	+	−	+	−	−	+	No	Prednisolone	Yes
		72	M	Right MHL	+	Right	+	−	−	+	Craniotomy	Corticosteroid	Yes
		67	F	Left MHL	+	−	+	−	−	+	Craniotomy	Prednisolone and azathioprine	Yes
Bravo et al. [18]	2007	53	M	CHL	+	−	+	ND	ND	+	Mastoidectomy and biopsy of dura matter	Methylprednisolone, antibiotics, and methotrexate	Yes
Lu et al. [19]	2009	51	F	Right MHL	+	Right	+	ND	ND	+	Biopsy of tentorium cerebelli and nasopharynx	Corticosteroid and antibiotics	Yes
Kobayakawa et al. [20]	2010	70	F	Left CHL	+	−	ND	ND	ND	+	Mastoidectomy and tympanoplasty	Corticosteroid and antibiotics	Recurrence
*Hasegawa et al. [21]	2012	48	M	Bilateral MHL	+	Left	+	+	−	+	Pharyngeal biopsy	Prednisolone and cyclophosphamide	Yes
Keshavaraj et al. [23]	2012	40	F	MHL	+	−	+	−	−	+	Craniotomy	Corticosteroid	Yes
*Mori et al. [22]	2013	63	M	Bilateral MHL	+	Bilateral	+	+	−	+	ND	Antibiotics, corticosteroid and immunoglobulin	Recurrence
*Saito et al. [24]	2014	72	F	Bilateral MHL	+	ND	+	+	−	+	ND	Corticosteroid and immunosuppressant	Yes
Okada et al. [25]	2015	63	M	Left MHL	+	−	+	−	−	+	Mastoidectomy and tympanostomy tube insertion	Antibiotics and voriconazole	Yes
Fujimoto et al. [26]	1993			4 patients were reported with HP and OM. They showed multiple cranial nerves involvement. Of which, 3 patients received corticosteroid and the symptoms improved									
*Yokoseki et al. [27]	2014			21 patients were reported with ANCA-related HP. More than 75% patients had hearing loss. Profound hearing impairment was common. Steroid plus immunosuppressant was very effective									
*Harabuchi et al. [8]	2017			They performed a retrospective analysis of 235 patients classified as OMAAV in Japan. 28% of patients were reported with HP. MPO-ANCA positivity was common. 60% patients received steroid plus immunosuppressant, and nearly half of patients had disease relapse									

*MHL* indicates mixed hearing loss, *CHL* conductive hearing loss, *SHL* sensorineural hearing loss, *ND* undetermined or unknown results, + positive findings, − no positive findings

*20 studies described the patients with HP and OM, and of these, 6 were involved in ANCA-related HP and OM

## Discussion

### Diagnosis

Patients were diagnosed with ANCA-related HP or OMAVV if serum MPO–ANCA and/or PR3-ANCA titers were elevated or if ANCA-related disease (such as GPA, MPA or EGPA) co-occurred with HP or OM. Similarly, patients were diagnosed with ANCA-related HP and OM if AAV patients co-occurred with HP and OM. Consequently, patients with ANCA-related HP and OM could be diagnosed based on established criteria for AAV, HP, and OMAAV. These include histopathology consistent with AAV or positivity for serum MPO–ANCA and/or PR3-ANCA; intractable OM with effusion or granulation that will not respond to antibiotics and tympanic ventilation tubes, and exclusion of other types of OM, such as bacterial OM, choleastoma, tuberculosis, and neoplasms. In addition, diagnosis was made if enhancement of dural mater on contrast-enhanced MRI or chronic inflammatory changes on dural biopsy were noted. Efficacy of corticosteroid and an immunosuppressant such as cyclophosphamide was considered diagnostic for ANCA-related HP and OM.

### Clinical presentation

Due to effusion or granulation in the middle ear, progressive conductive hearing loss may occur. Then, sensorineural hearing loss may gradually occur as inner ear disturbances progress. Therefore, three types of hearing impairment may be found: conductive, mixed, and sensorineural hearing loss. Hearing loss was reversible for some cases after therapy, but complete deafness is often difficult to reverse. Profound hearing impairment was evident for patients with OMAAV and 64% of ANCA-positive patients had severe hearing loss [27]. Although more than half of patients had otorrhea, no bacterial pathogens were noted with otorrhea cultures or ear exudate analysis. Cranial nerve involvement may occur for patients with ANCA-related HP and/or OMAAV. For example, 30–50% of those patients had peripheral facial palsy during the clinical course [8]. Furthermore, more diffuse symptoms such as headache and seizures may occur as disease progresses, and severe headache is the most common symptom of HP. This is regarded as an important factor associated with HP occurrence in AAV or OMAAV patients.

### Serum ANCA status

Although histopathological identification of necrotizing vasculitis is the key to diagnosing AAV, when pathology cannot be certain or specimens cannot be easily obtained, misdiagnosis may occur [4, 5]. For example, a middle ear specimen often showed a lower positive rate compared to the other specimen taken from nose or lung which may lead to misdiagnosis [28]. Serum ANCA reactivity and clinical symptoms maybe the most important findings for diagnosing AAV. Elevation of ANCA titers is thought to be a highly sensitive and specific serological index for AAV such as GPA, MPA and EGPA [4, 29]. But not all the patients with AAV have positive results on serologic testing for ANCA, for example, for patients with ANCA-negative AAV, they may have ANCA that cannot be detected with current methods or may have ANCA of as yet undiscovered specificity [5]. Moreover, ANCA status may convert to MPO-ANCA and/or PR3-ANCA positivity as disease progresses. ANCA status provides us an important index for documenting therapeutic effects. Patients initially ANCA-positive may revert to ANCA-negative status after therapy, suggesting disease improvement. In contrast, the reappearance of ANCA titers can indicate a relapse.

In previous studies, ANCA status was reported to be influenced by regional factors. For example, MPO–ANCA positivity is more common in China, Japan and Korea, whereas PR3-ANCA is more frequent in Europe and the US [27, 30–34]. MPO–ANCA positivity was more frequent for patients with ANCA-related HP and OM, likely because most relevant reports were from Japanese scholars in this review. ANCA status may be influenced by environmental factors and genetics. A recently published genome-wide association study confirmed that PR3-ANCA was associated with the human major histocompatibility complex (MHC) class II isotype HLA-DP, the gene encoding α-1-AT (SERPINA1) and the gene encoding PR3 (PRTN3), whereas MPO-ANCA was associated with MHC class II isotype HLA-DQ [35].

Clinical features vary by ANCA status. Patients with PR3-ANCA positive OM have granulomatous formation or effusion in the middle ear, whereas MPO–ANCA positive OM predominantly presents as OM with effusion [7, 8, 27]. Most patients with MPO–ANCA positive HP have the CNS-limited form and a less severe phenotype compared with patients with PR3-ANCA positive HP [27, 36, 37]. In addition, Studies suggest that PR3-ANCA-positive subjects had greater involvement of brain parenchyma, renal parenchyma and lung compared with MPO–ANCA positive subjects [38]. However, whether patients with PR3-ANCA positivity have more severe neurological damage and more severe disease compared with patients who are MPO–ANCA positive is still unclear, and the association between ANCA status and disease severity is not clear either.

### Radiology

**Fig. 2:**
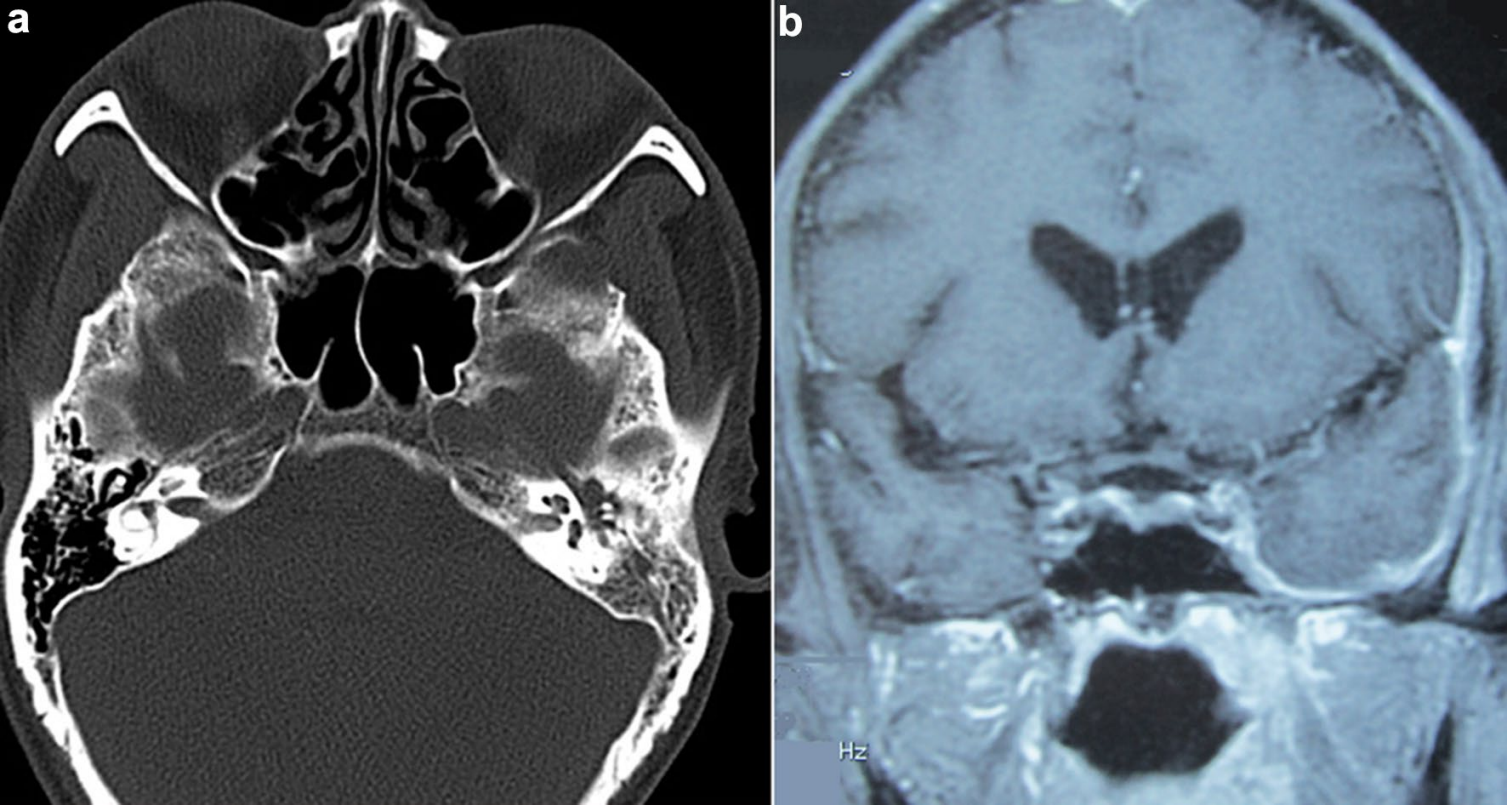
Imaging findings of a patient with ANCA-related HP and OM. **a** Axial CT: the left middle ear and mastoid cavities are filled with soft-tissue mass. **b** Coronal MRI: dural thickness of the left temporal lobe and tentorium cerebella

Imaging may be used to identify OM and fibrotic meningeal lesions of HP, analyze adjacent structures, and evaluate curative effects of ANCA-related HP and OM. Computed tomography (CT) of the temporal bones is useful for diagnosing OM, and the middle ear and mastoid cavities filled with soft tissue material are common (Fig. 2). Magnetic resonance imaging (MRI) offers some advantages over CT for the assessment of meningeal lesions. Contrast-enhanced MRI of the brain or spine can be used to diagnose HP, and gadolinium-enhanced T1-weighted MRI reveals thickening of the dural mater, especially when it is difficult to obtain specimen or make a positive diagnosis of HP with histopathology. Moreover, axial T2-weighted MRI may be used to observe inflammation of the middle ear and mastoid cavities.

There have been reported significant overlaps in the distribution pattern of dural enhancement among patients with MPO–ANCA positive HP, PR3-ANCA positive HP, immune-mediated, and idiopathic HP, and there is no consensus on the correlation between dural enhancement pattern and HP etiology [27, 39]. But fortunately, recent imaging studies indicated that most patients with MPO–ANCA positive HP have the CNS-limited form and less frequent leptomeningeal or parenchymal involvement than those with PR3-ANCA-positivity [36, 37]. However, the association between ANCA status and dural enhancement pattern of HP and its pathological mechanism remain to be further studied.

### Treatment

Treatment of AAV includes remission induction and maintenance. The European League against Rheumatism (EULAR) recommended that AAV should be treated according to severity and organ involvement with prednisolone and cyclophosphamide used for remission and induction for patients with organ-threatening AAV. Prednisolone and methotrexate can be used for remission and induction of patients with AAV with non-organ threatening or non-life threatening disease [40]. Corticosteroid and cyclophosphamide are more effective than corticosteroid alone, offering long-term remission, better hearing outcomes, and survival for patients with ANCA-positive HP or OMAAV [7, 8, 27, 30, 41]. Therefore, initial treatment with corticosteroid and an immunosuppressant such as cyclophosphamide should be recommended for ANCA-positive HP and OM, moreover, it should be also used in the event of a recurrence [5, 42, 43]. Thus, it can be seen that the treatment for this subgroup of patients with AAV is different from other AAV patients with non-organ threatening disease. Furthermore, the treatment of this disease is different from that of intractable OM or HP caused by other etiologies. For example, for patients with idiopathic HP, concomitant OM and HP, or IgG4-related HP, most of the published reports reveal a preference for corticosteroid treatment, followed by the addition of an immunosuppressant at relapse [6, 14, 17, 44].

Therapy has improved for AAV treatment, but most treatment recommendations are empirical and require more study. For example, immunosuppressant selection and dose adjustment is based on patient age, ANCA status, and disease severity but these need more scrutiny. In addition, immunosuppressive regimens may be associated with gonadal toxicity, diabetes, thromboembolism, and cardiovascular disease. A recent study suggested that death due to adverse events in the first year of treatment was three times more likely than from vasculitis itself [45, 46]. Therefore, new highly selective immunosuppressant drugs that have little toxicity are needed.

### Pathogenesis

Although the etiology and mechanism of ANCA-related HP plus OM is unclear, increasing awareness of AAV contributes to understanding ANCA-related HP and OMAAV due to a common etiologic basis. AAV is considered to be an autoimmune disease associated with ANCA. In vitro and animal models suggest that ANCA may contribute to the formation of small vessel vasculitis [47]. MPO and PR3 specific ANCA can activate neutrophils and monocytes via interactions with target antigens translocated from the lysosomal compartment to the cell surface due to triggers such as infectious agents, cytokines, and chemokines. Activated neutrophils not only release free oxygen radicals and lytic enzymes but also trigger endothelial activation [48, 49]. Although the role of ANCA is generally accepted, studies confirm that normal individuals without vasculitis were positive for ANCA [50]. In addition, TH1-predominant granulomatous lesions were found in patients with ANCA-positive HP, suggesting that ectopic lymphoid neogenesis may play a role [27]. In summary, AAV may involve molecular signaling pathways with positive or negative feedback loops.

**Fig. 3:**
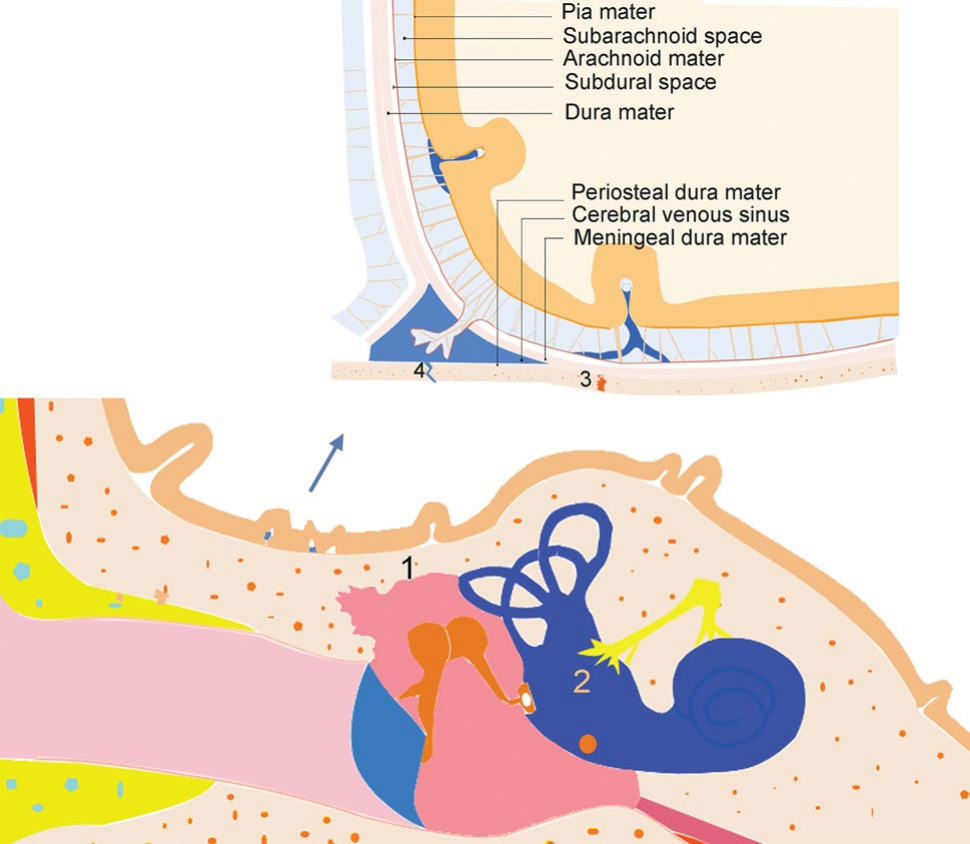
The anatomy of the middle ear and the dura mater. 1 tegmen tympani, 2 inner ear, 3 temporal bone sutura or fissure, 4 intracranial venous sinus

Whether a potential relationship exists between HP and OM occurring in AAV patients is unclear. Some studies suggest a relationship that is associated with middle ear and dural mater anatomy (Fig. 3) [7, 17, 19, 27]. For example, small vasculitis leading to granulation or effusion in the middle ear may spread to the dural mater and lead to secondary HP via several ways: damaged tympanicum tegmentum; temporal bone sutura or fissure; the inner ear including the labyrinth and vestibule; local circulation via venous return communicating with the middle ear and the dural mater in the middle and/or the posterior cranial fossa. In addition, small vasculitis or granulomatous lesions in the dural mater may also spread to the middle ear via the above ways and cause OM. In addition, the occurrence of ANCA-related HP and OM may be thought of as distinct but synchronous events.

## Conclusions

**Fig. 4:**
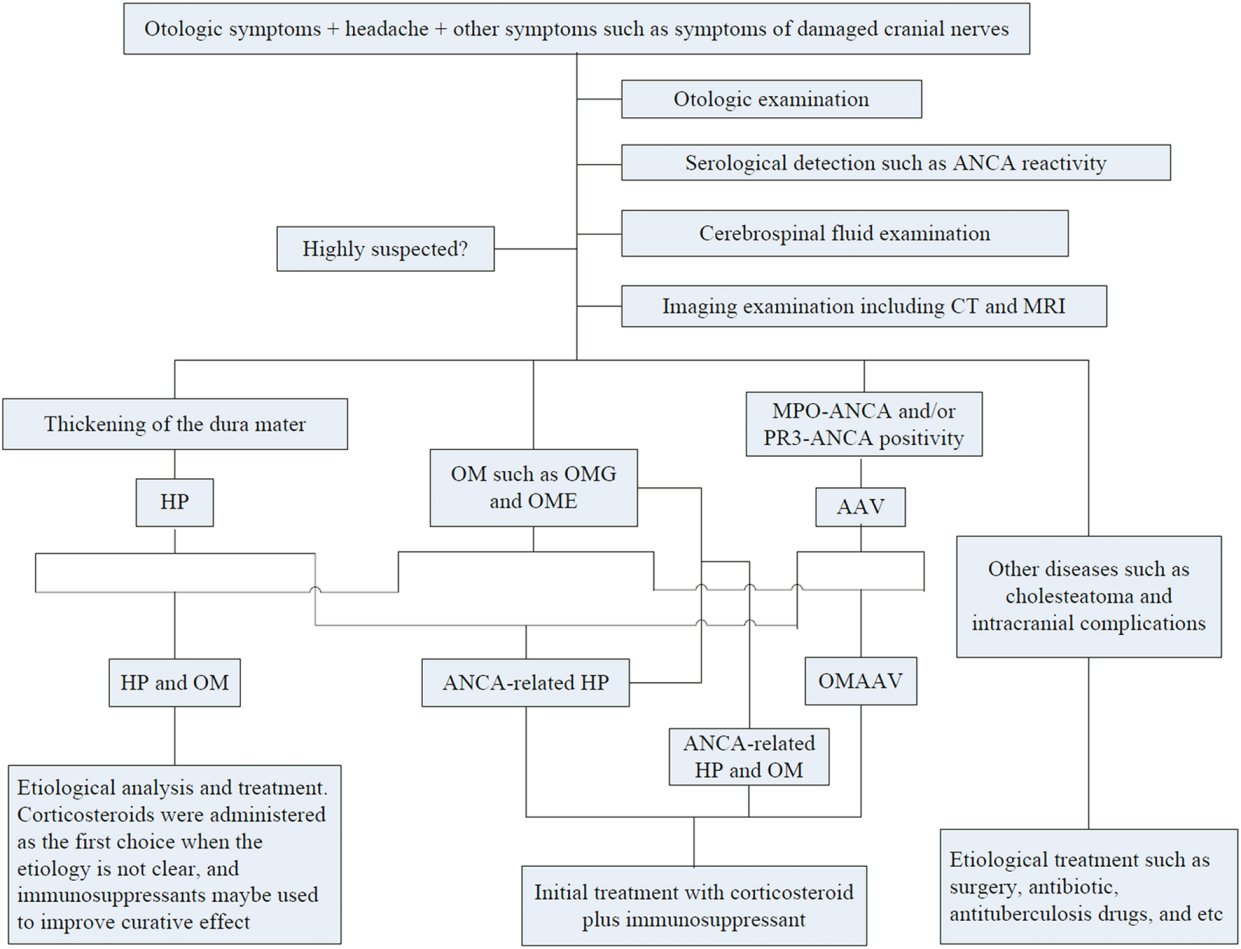
Schematic overview of the diagnosis and treatment

Here, we summarize clinical features, diagnosis, and treatment of ANCA-related HP and OM and its etiology (Fig. 4). Early hearing loss may be reversible due to cochlear homeostatic function, so rapid diagnosis and treatment are critical for recovery of auditory function [7, 8, 27, 51]. In addition, we should carefully screen out the potential cases with ANCA-related HP and OM in a population with intractable OM, HP, or AAV, and make appropriate treatment. Initial treatment with corticosteroids and an immunosuppressant should be recommended. However, due to an increased possibility of relapse, close follow-up with a hearing test, MRI, and ANCA titers, is needed. Furthermore, prevention and management of the adverse effects associated with immunosuppressive therapy is very important for patients with ANCA-related HP and OM.
